# Oral faecal microbiota transplantation for the treatment of *Clostridium difficile*-associated diarrhoea in a dog: a case report

**DOI:** 10.1186/s12917-018-1754-z

**Published:** 2019-01-07

**Authors:** Koji Sugita, Nanako Yanuma, Hikaru Ohno, Kaho Takahashi, Koji Kawano, Hidetoshi Morita, Keitaro Ohmori

**Affiliations:** 1 Cooperative Department of Veterinary Medicine, Faculty of Agriculture, Tokyo University of Agriculture and Technology, 3-5-8 Saiwaicho, Fuchu, Tokyo, 183-8509 Japan; 2 Sugita Animal Hospital, 3-55-10 Shinshiraoka, Shiraoka, Saitama, 349-0212 Japan; 3 Primo Animal Hospital Tokyo Animal Allergy Medical Center, 1-28-7 Shakujiimachi, Nerima, Tokyo, 177-0041 Japan; 4 Graduate School of Environmental and Life Science, Okayama University, 1-1-1 Tsushimanaka, Kita, Okayama, Okayama 700-8530 Japan

**Keywords:** *Clostridium difficile*, Diarrhoea, Dog, Oral faecal microbiota transplantation

## Abstract

**Background:**

Successful clinical outcomes of faecal microbiota transplantation (FMT) for recurrent *Clostridium difficile* infection have been reported in humans and a marmoset. However, it has been unclear whether oral FMT was effective for the treatment of *C. difficile*-associated diarrhoea in dogs.

**Case presentation:**

An 8-month-old, intact male French bulldog was presented with a 4-month history of intermittent large bowel diarrhoea. Physical and clinical examinations did not identify any specific causes for diarrhoea. Real-time PCR analysis and immunochromatography detected *C. difficile* antigen and toxin A&B genes and proteins in a faecal sample. Based on these findings, diarrhoea in the dog was considered to be induced by *C. difficile*-associated colitis. The dog was treated with oral FMT, in which a faecal solution obtained from a healthy beagle was orally administered to the subject. Stool consistency and frequency and faecal blood and mucus became normal 2–3 days after oral FMT, and real-time PCR analysis and immunochromatography was negative for *C. difficile* antigen and toxin A&B genes and proteins. No adverse events were observed.

**Conclusion:**

The present case report demonstrated that oral FMT was an effective treatment for *C. difficile*-associated diarrhoea in a dog. The findings in this report provide a rationale to evaluate clinical efficacy of oral FMT for other gastrointestinal diseases in dogs.

## Background

*Clostridium difficile* is the most common cause of antibiotic-associated pseudomembranous colitis and induces severe and recurrent diarrhoea, especially in hospitalized human patients [1]. *C. difficile* is also associated with enterocolitis and diarrhoea in animals including dogs [2] and marmosets [3]. Metronidazole is an effective antibiotic for the treatment of *C. difficile* infection (CDI) in humans [1] and animals [2]. However, recurrent CDI after treatment with antibiotics including metronidazole has become a clinical problem in human patients [1].

Faecal microbiota transplantation (FMT) is a treatment option performed by introducing faecal microbiota obtained from a healthy donor into the gastrointestinal (GI) tract of a recipient [4, 5]. Successful clinical outcomes of FMT for recurrent CDI have been reported in humans [5–7] and a marmoset [3]. However, it has been unclear whether FMT was effective for the treatment of *C. difficile*-associated diarrhoea in dogs. Here, we report persistent recovery from *C. difficile*-associated diarrhoea in a dog after oral FMT without any adverse events.

## Case presentation

An 8-month-old, 11.0-kg, sexually intact male French bulldog was presented on day 1 with a 4-month history of intermittent diarrhoea and a 7-day history of focal seizures that had been observed almost every day for 7 days. Stool consistency had been very soft to watery, and stool frequency had been > 7 times/day. Blood and mucus had been observed in the faeces. Thus, diarrhoea was considered to be induced by colitis. Four months prior to the current presentation, a faecal sample of the dog was subjected to real-time PCR analysis (IDEXX Laboratories, Inc., Tokyo, Japan) for *Cryptosporidium* spp., *Giardia* spp., *Clostridium perfringens* α toxin, *Clostridium difficile* toxin A&B, *Campylobacter jejuni*, *Campylobacter coli*, *Salmonella* spp., Canine parvovirus type 2, canine distemper virus and canine enteric coronavirus genes by a veterinary practitioner; a positive reaction for *Campylobacter jejuni* was detected in the analysis. The dog was treated with tylosin (Tylan, Eli Lilly Japan K.K., Kobe, Japan; 10 mg/kg PO, q12h) for 7 days by a veterinary practitioner; however, stool conditions did not improve. Administration of an antidiarrhoeal (Diabuster, Kyuritsu, Tokyo, Japan; 1 tablet PO, q12h) containing berberine tannate, bismuth subnitrate, geranium herb, nutgalls and scopolia extract, and an antiflatulent (Bioymbuster, Kyuritsu, Tokyo, Japan; 1 tablet PO, q12h) containing *Bacillus coagulans, Bifidobacterium longuin*, *Lactobacillus acidophilus*, *Streptococcus faecalis* and pancreatin, improved stool conditions. However, once these drugs were discontinued, the diarrhoea recurred.

On day 1, physical and clinical examinations, including a complete blood count (CBC), a serum biochemical analysis, radiography, an abdominal ultrasound and faecal examination, did not reveal any specific causes for chronic diarrhoea and focal seizures. A faecal sample was subjected to real-time PCR analysis (IDEXX Laboratories, Inc.) to investigate an infectious cause of diarrhoea. Meanwhile, the dog was administered erythromycin (Erythromycin, Sawai Pharmaceutical, Osaka, Japan; 10 mg/kg PO, q12h) for 14 days based on the positive result for *C. jejuni* infection 4 months earlier.

On day 2, real-time PCR analysis of a faecal sample collected on day 1 was found to be positive for *C. difficile* toxin A&B genes and negative for other pathogens. The presence of *C. difficile* antigen and toxin A&B proteins in a faecal sample collected on day 1 was also confirmed by an immunochromatographic test kit (Techlab *C. Diff* Quick Chek Complete, Alere, Chiba, Japan).

In the follow-up visit on day 16, stool conditions did not improve after administration of erythromycin in the dog. Based on the clinical and investigative findings, diarrhoea in the dog was considered to be induced by *C. difficile*-associated colitis. Treatment with metronidazole was proposed; however, the owner rejected this treatment because of the potential for metronidazole-induced neuropathy. To investigate the cause of focal seizures, computed tomography and magnetic resonance imaging were performed. Mild ventriculomegaly was detected in the brain of the dog on imaging, but it was unclear whether the lesion was related to the seizures. After initiating treatment with zonisamide (Consave, DS Pharma Animal Health, Osaka, Japan; 10 mg/kg PO, q12h), the seizure frequency decreased.

On day 25, the dog still had large bowel diarrhoea. Real-time PCR analysis and immunochromatography confirmed that *C. difficile* antigen and toxin A&B genes and proteins were still positive in a faecal sample collected on day 25. Therefore, instead of treatment with metronidazole, oral faecal microbiota transplantation (FMT) was performed after obtaining written informed consent from the owner. This treatment was approved by the Research Ethics Committee of Tokyo University of Agriculture and Technology. Fresh faeces were collected from a 9-year-old, 11.0-kg, sexually intact healthy male beagle maintained for research purposes. The healthy dog was housed in a cage and fed a commercial diet (Science Diet Adult, Hill’s-Colgate Ltd., Tokyo, Japan) once daily. Water was provided ad libitum. Physical and clinical examinations, including a CBC, a serum biochemical analysis, radiography, an abdominal ultrasound and faecal examination, did not find any abnormalities in the healthy dog, and real-time PCR analysis of a faecal sample did not detect any pathogens. Immediately after faecal collection, approximately 60 g of faeces was dissolved in 50 mL of tap water. The faecal solution was filtered through a medical gauze pad twice. A total of 30 mL of a filtered faecal solution was obtained and orally administered to the recipient dog using a syringe.

Stool consistency became normal, and stool frequency was reduced to 4–5 times/day 2–3 days after oral FMT. Faecal blood and mucus were not observed after oral FMT. Real-time PCR analysis of a faecal sample collected at 7 days after oral FMT (day 32) was negative for *C. difficile* toxin A&B genes. Further real-time PCR analysis of faecal samples collected on days 61 and 149 confirmed that *C. difficile* toxin A&B genes were still negative. The absence of *C. difficile* antigen and toxin A&B proteins was also verified in the faecal samples by an immunochromatographic test kit after oral FMT. In addition, diarrhoea did not recur after oral FMT and further medications were unnecessary. Stool conditions are still normal on day 190.

## Discussion and conclusions

The present case report demonstrated that *C. difficile* antigen and toxin A&B genes and proteins turned into negative, and stool consistency and frequency and faecal blood and mucus became normal after oral FMT in a dog with large bowel diarrhoea. Successful clinical outcomes of FMT for recurrent CDI have been reported in humans [5–7] and a marmoset [3]. These findings collectively suggest that correction of gut microbiota with FMT can be a useful treatment option for *C. difficile*-associated diarrhoea across animal species.

The pathogenesis of CDI is well established in humans, and involves toxin production by colonic *C. difficile* and depletion of non-*C. difficile* colonic microbiota [5]. However, it is still controversial whether *C. difficile* plays a pathological role in the development of diarrhoea in dogs [8]. *C. difficile* has been isolated both from the faeces of diarrheic dogs and those of healthy, non-diarrheic dogs, with various incidence rates depending on sample populations [9–13]. Several studies suggested a significant association between the presence of *C. difficile* toxins in faeces and canine diarrhoea [9–11]. An outbreak of *C. difficile*-associated disease was also reported in a small animal veterinary teaching hospital [14]. In contrast, a previous study failed to reproduce CDI in healthy adult dogs after administration of *C. difficile* with or without antibiotics [15]. In the present report, real-time PCR analysis and immunochromatography detected *C. difficile* antigen and toxin A&B genes and proteins in a faecal sample. Physical and clinical examinations did not identify any other causes for chronic large bowel diarrhoea. In addition, *C. difficile* antigen and toxin A&B genes and proteins became negative after oral FMT, and diarrhoea did not recur despite no further pharmacological treatment. Based on these clinical and molecular findings, diarrhoea in this dog was considered to be induced by *C. difficile*-associated colitis.

FMT can be performed via the upper or lower GI tract [5]. Theoretical guidelines for FMT have been recently proposed in dogs and cats [16]. In a recent study that reported the clinical efficacy of FMT for puppies with canine parvovirus infection [17], faecal suspension was infused into the proximal portion of the rectum in puppies by retention enema. Since clinical data of FMT are very limited in veterinary medicine, there is no consensus regarding the appropriate method of faecal administration in dogs. In the present case, FMT was performed by oral administration of faeces diluted with tap water. Oral FMT was also shown to be effective for the treatment of CDI in a marmoset [3], whereby faeces were mixed with the marmoset’s usual food and fed to the subject. In human patients with CDI, there was a report of cases refractory to lower GI delivery that responded to FMT via oral frozen faecal capsules [18]. Oral FMT is much easier than other methods of FMT, such as FMT with endoscopy, nasogastric/nasoenteric tubes and retention enema. Although potential advantages and disadvantages exist for FMT via the upper or lower GI tract, the present report suggests that oral administration of a faecal solution can be a useful FMT method in dogs.

The recipient dog in this report did not show any adverse events after oral FMT until day 190. The recipient dog was orally administered a fresh faecal solution prepared from a healthy beagle that did not have any abnormalities on physical and clinical examinations and any faecal pathogens.

Metronidazole is reported to be an effective antibiotic for the treatment of CDI in dogs [2] and humans [1]. However, in vitro analyses showed metronidazole-resistant strains in faeces of dogs [19, 20]. In human patients, as many as 20% of CDI cases treated with antibiotics including metronidazole was reported to recur [5] and a target for FMT. In this report, because metronidazole was not administered at the owner’s request, it is unclear whether *C. difficile* detected in the dog was metronidazole-resistant. Previous studies have shown that FMT was effective for the treatment of antibiotics-resistant recurrent CDI in humans, with cure rates of > 80% [5–7], and a marmoset [3]. To evaluate the effect of FMT for metronidazole-resistant recurrent CDI in dogs, further studies are required.

The mechanism underlying FMT is considered to be the reestablishment of the normal gut microbiota as a host defense against CDI [21]. A previous study showed that faecal microbiota in human patients with CDI had a lower bacterial diversity than healthy humans; in these patients, FMT improved the microbiota diversity [7]. In the current report, faecal microbiota was not analyzed in the dog with CDI. To clarify the mechanism by which oral FMT exerts a treatment effect on CDI in dogs, it is necessary to compare faecal microbiota in canine patients with those in healthy dogs, and to evaluate the microbiota in canine patients before and after oral FMT.

In conclusion, the present report revealed that oral FMT was an effective treatment for *C. difficile*-associated diarrhoea in a dog. FMT can reintroduce normal microbiota from healthy individuals into patients, correcting the underlying dysbiosis in the gut. Previous studies have reported dysbiosis was associated with acute and chronic GI diseases in dogs, including idiopathic inflammatory bowel disease [22–24]. The findings in the present report provide a rationale to evaluate clinical efficacy of oral FMT for other GI diseases in dogs.

## Abbreviations

*C. difficile*: *Clostridium difficile*
CBC: complete blood count
CDI: *Clostridium difficile* infection
FMT: Faecal microbiota transplantation
GI: Gastrointestinal
